# The Assessment of IL-21 and IL-22 at the mRNA Level in Tumor Tissue and Protein Concentration in Serum and Peritoneal Fluid in Patients with Ovarian Cancer

**DOI:** 10.3390/jcm10143058

**Published:** 2021-07-09

**Authors:** Aleksandra Mielczarek-Palacz, Celina Kruszniewska-Rajs, Marta Smycz-Kubańska, Jarosław Strzelczyk, Wojciech Szanecki, Andrzej Witek, Joanna Magdalena Gola

**Affiliations:** 1 Department of Immunology and Serology, Faculty of Pharmaceutical Sciences in Sosnowiec, Medical University of Silesia, 40-055 Katowice, Poland; apalacz@sum.edu.pl (A.M.-P.); mkubanska@sum.edu.pl (M.S.-K.); jarekstrzelczyk@o2.pl (J.S.); 2 Department of Molecular Biology, Faculty of Pharmaceutical Sciences in Sosnowiec, Medical University of Silesia, 40-055 Katowice, Poland; ckruszniewska@sum.edu.pl; 3 Department of Gynecology and Obstetrics, Faculty of Medical Sciences in Katowice, Medical University of Silesia, 40-055 Katowice, Poland; wszanecki@sum.edu.pl (W.S.); awitek@sum.edu.pl (A.W.)

**Keywords:** ovarian cancer, interleukin 21, interleukin 22

## Abstract

The aim of the analysis was for the first time to assess the expression of genes encoding IL-21 and IL-22 at the mRNA level in ovarian tumor specimens and the concentration of these parameters in serum and peritoneal fluid in patients with ovarian serous cancer. The levels of IL-21 and IL-22 transcripts were evaluated with the use of the real-time RT-qPCR. Enzyme-linked immunosorbent assay (ELISA) was used to determine the concentration of proteins. Quantitative analysis of IL-21 gene mRNA in the tumor tissue showed the highest activity in the G1 degree of histopathological differentiation and was higher in G1 compared to the control group. The concentration of IL-21 and IL-22 in the serum and in the peritoneal fluid of women with ovarian cancer varied depending on the degree of histopathological differentiation of the cancer and showed statistical variability compared to controls. The conducted studies have shown that the local and systemic changes in the immune system involving IL-21 and IL-22 indicate the participation of these parameters in the pathogenesis of ovarian cancer, and modulation in the IL-21/IL-22 system may prove useful in the development of new diagnostic and therapeutic strategies used in patients, which require further research.

## 1. Introduction

Ovarian cancer is one of the gynecological cancers with the worst prognosis. Both the number of cases and the mortality caused by this disease in the world are constantly increasing [1,2]. In patients, the most important prognostic factor is the stage of clinical advancement, which determines the therapeutic strategy. Unfortunately, this cancer is detected in stage III or IV in more than 70% of women. It is associated with an unfavorable prognosis and a low five-year survival rate. It is caused by asymptomatic tumor growth, lack of characteristic clinical symptoms in the initial stage of the disease and diagnostic tests helpful in early diagnosis [3,4]. Currently conducted research indicates that the protein intercellular mediators—cytokines—play an important role in the formation and development of ovarian cancers. Cytokines interact with cells through characteristic surface receptors that transmit a signal to the interior of the cell. Altered expression of cytokines and their receptors in cancer cells affects the interaction in the tumor microenvironment, which may induce an anti-cancer response, promote tumor growth, participate in invasion and metastasis, and in immunosuppression [5,6].

Recent studies indicate that interleukin 21 (IL-21) and interleukin 22 (IL-22) play an important role in the pathogenesis of cancer and cancer therapy [7,8,9,10,11,12]. Winkler et al. showed, that Th17 cell sub-populations with expression of IL-21 and/or IL-22 infiltrate the tumor tissue of ovarian cancer [7]. Interleukin-21 is a cytokine secreted mainly by activated CD4 + T cells, including T helper 17 (Th17) and T follicular helper (Tfh) cells and NKT cells [8]. IL-21 is bound to the IL-21 receptor which contains the gamma chain [9]. It has an immunoregulatory function, has the ability to both promote and inhibit the immune response and induce an anti-cancer response, mainly by activating Tc lymphocytes, therefore, attempts are still being made to use it in cancer immunotherapy [10,11,12]. Studies have shown the effectiveness and safety of the therapy with the use of recombinant IL-21 in advanced melanoma, renal cell carcinoma, and non-Hodgkin’s B cell lymphoma [13]. Subcutaneous administration of IL-21 augmented tumor regression and increased tumor infiltration by CD8 + T cells [11]. IL-21 can be also combined with other immunotherapies [14].

Interleukin-22 is a cytokine synthesized by lymphocyte T helper (Th) cells, including their Th17 and Th22 subpopulations as well as NKT lymphocytes, NK cells, and nonspecific lymphoid cells [15]. It is a pro-inflammatory cytokine, it induces the production of acute phase proteins and participates in the pathogenesis of many diseases, including cancer. Interestingly, IL-22 has also been shown to induce multiple cytoprotective mechanisms, participating in tissue regeneration following inflammatory lesions [16]. IL-22 is bound to the heterodimer receptor complex of IL–10R2 and IL-22R1 [17] and, by activating numerous signaling pathways, it influences such processes as cell survival, proliferation, migration, and angiogenesis [17]. These processes are crucial for the development of cancer. Protopsaltis et al. demonstrated that IL-22 directly stimulates angiogenesis through activation of the ERK and Stat3 pathways, and blockade of IL-22 inhibits tumor growth [18]. Interestingly, cancer cells release IL-1, which stimulates IL-22 production by memory T cells [17].

The analyses conducted so far on the evaluation of IL-21 and IL-22 in ovarian cancer have been interesting and induce further research. It has been shown that the determination of IL-21 with other cytokines, for example, IL-17a, may find clinical application as prognostic and therapeutic biomarkers [19]. Interleukin-22 has been shown to be an important factor in the ovarian cancer tumor microenvironment and may find application as a potential therapeutic target and/or biomarker [20]. Its prognostic value in other cancers has also been established, including pancreatic cancer [21] and hepatocellular carcinoma [22].

So far, no studies have been conducted to assess both the expression of IL-21 and IL-22 in tumor tissue, as well as the concentration of these cytokines in the serum and peritoneal fluid in women with ovarian cancer, and considering the fact that understanding the role of these parameters in the pathogenesis of ovarian cancer may be useful in developing new diagnostic and therapeutic regimens in patients with ovarian cancer in the future, the aim of the study was: assessment of IL-21 and IL-22 at the mRNA level in ovarian cancer tissue, analysis of the concentration of IL-21 and IL-22 in the serum and peritoneal fluid of patients with ovarian cancer, and determining whether there is a relationship between the concentration of IL-21 and IL-22 and the degree of histological differentiation of the cancer.

## 2. Materials and Methods

The study group included 26 women, aged 41 to 82 (mean age: 62.80 ± 11.21 years) with the diagnosed ovarian cancer with the Cystadenocarcinoma papillare serosum IIIC (7 had G1, 5 had G2, and 14 had G3 staging) Staging employed the criteria recommended by the International Federation of Gynecology and Obstetrics (FIGO). The diagnosis of tumors was done on the basis of clinical symptoms, results of gynecological and histopathological examination and laboratory tests. The women qualified to the studied group were clinically diagnosed with ovarian tumor confirmed with a histopathological examination. Other coexisting disorders of the reproductive organs and autoimmune diseases were excluded. None of the examined women used pharmacological treatment in the last three months. The research was conducted on women hospitalized at the Clinical Department of Gynecology and Obstetrics of the University Clinical Center in Katowice and the Department of Gynecology and Obstetrics with the Department of Pathology of Pregnancy and Gynecological Oncology of the Provincial Specialist Hospital in Częstochowa. Serum, peritoneal fluid, and tumor tissue were the material examined in all women. In the study group, blood was taken from women after establishing clinical diagnosis, before surgery. Blood was taken in the morning from the cubital vein, to a clot tube, in order to obtain the serum. Thirty minutes after taking the blood, it was centrifuged at 1500× *g* for 15 min. The serum obtained in this manner was kept in small portions at a temperature of −80 °C until the tests. Tumor tissue intended for molecular assessment was collected during the planned surgery and frozen at −80 ° C until the tests were performed. Peritoneal fluid was collected during laparoscopy for bacteriological examination, and then centrifuged at 2000 rpm for 10 min at 4 °C, and the obtained supernatant was partitioned and frozen at −80 °C until the remaining determinations were made.

The control group consisted of six women aged between 40–77 (mean age: 61.83 ± 12.48 years) who have been diagnosed with a benign lesion (Cystadenoma serosum). The concentration of antigen CA125 did not exceed 35 U/m in these patients. In all the women, the blood serum and tissue samples were the research material.

Total RNA was extracted from tissue samples with the use of TRIzol reagent (Thermo Fisher Scientific, Waltham, MA, USA), according to the manufacturer’s instructions. Each tissue sample was placed in a tube containing TRIzol, and then homogenized using a Polytron^®^ homogenizer (Kinematica AG, Malters, Switzerland). RNA extracts quantitative assessment was performed with the use of nanospectrophotometer MaestroNano MN-913 (MaestroGen Inc., Hsinchu City, Taiwan) and 2100 Bioanalyzer (Agilent Technologies, Inc. Santa Clara, CA, USA) (Figure S1, supplementary materials). The levels of IL-21 and IL-22 transcripts were evaluated with the use of the real-time RT-qPCR. The quantitative analysis was carried out using LightCycler^®^ 480 System (Roche, Basel, Switzerland) and GoTaq^®^ 1-Step RT-qPCR System (Promega, Madison, WI, USA), according to manufacturers’ instructions. Amplification was performed using previously described oligonucleotide primers [23,24] and commercially available standards of β-actin cDNA (TaqMan™ DNA Template Reagents; Thermo Fisher Scientific, Waltham, MA, USA). All samples were tested in triplicate. The mRNA copy numbers of the gene examined were recalculated per 1 µg of the total RNA. Melting curve analysis and agarose gel electrophoresis were used to confirm the specificity of amplification and the absence of primer dimers (Figures S2–S4, supplementary materials). Enzyme-linked immunosorbent assay (ELISA) was used to determine the concentration of the studied parameters. The following kits were used for this purpose: IL-21 Human Interleukin-21 ELISA and Human Interleukin-22 (BioVendor–Laboratorni medicina a.s., Brno, Czech Republic). Interleukin-21 Human ELISA, Sandwich ELISA, Biotin-labelled antibody, Calibration Range 78–5000 pg/mL, limit of detection 20.0 pg/mL and Interleukin-22 Human ELISA, Sandwich ELISA, Biotin-labeled antibody, Calibration Range 31.3–2000 pg/mL, limit of detection 5.0 pg/mL.

All the women who participated in the study consented to conducting the research. The approval of the Ethics Committee of the Medical University of Silesia in Katowice was obtained.

The obtained results were statistically analyzed using the Statistica 13.3 software (StatSoft Polska Sp. z o.o., Cracow, Poland). The normality of the distribution of the studied variables was checked using the Shapiro–Wilk test. The median and quartile range were determined for the parameters tested, and the obtained results were compared using the Mann–Whitney test. Correlations were tested by Spearman’s rank correlation test and presented as correlation coefficient (*r*).

## 3. Results 

**Figure 1:**
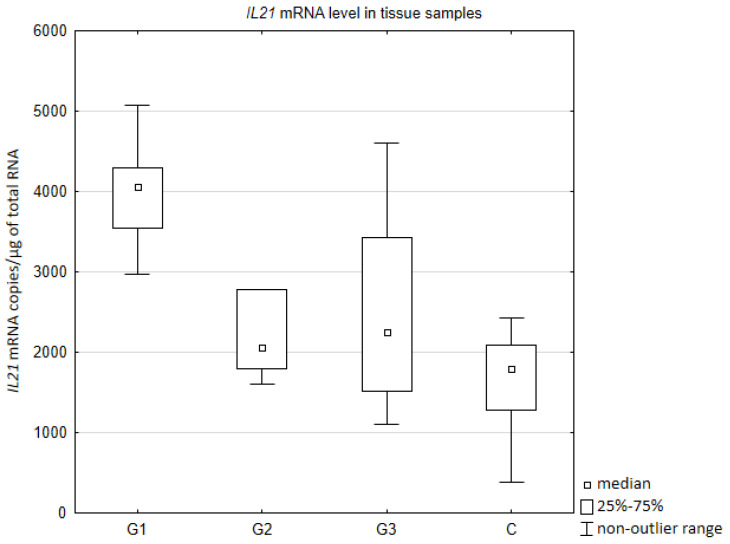
Number of copies of IL21 mRNA/µg of total RNA in tissue samples depending on the degree of ovarian cancer differentiation G1 (well differentiated), G2 (moderately differentiated), G3 (poorly differentiated) compared to the control group (C).

Quantitative assessment of IL21 mRNA in tissue samples showed the highest gene activity in the degree of differentiation G1 (Figure 1). The expression of IL21 gene was higher comparing to control group (*p* = 0.002415), G2 (*p* = 0.033161), and G3 (*p* = 0.020397). No statistically significant difference was found between G2, G3, and a control group. Moreover, expression of IL21 mRNA did not differ between G2 and G3.

Quantitative assessment of IL22 mRNA in tissue samples did not show statistically significant difference comparing to a control group. Moreover, expression of IL22 mRNA did not differ between degrees of differentiation G1, G2, and G3. Median values of the number of copies of IL21 mRNA/µg of total RNA in tissue samples were as follows: G1: Me = 6660, G2: Me = 5065, G3: Me = 7820, C: Me = 8555.

**Figure 2:**
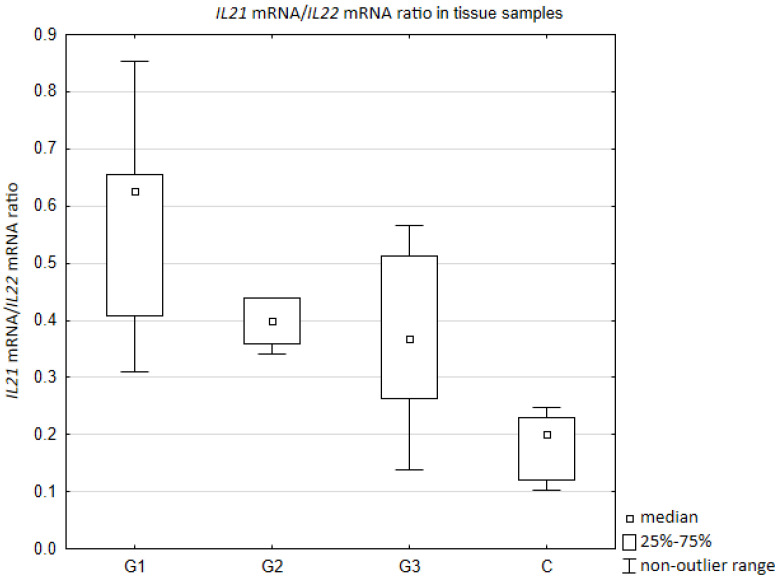
Il-21/IL-22 mRNA ratio in tissue samples depending on the degree of ovarian cancer differentiation G1 (well differentiated), G2 (moderately differentiated), G3 (poorly differentiated) compared to the control group (C). Ratio was calculated based on mRNA copies/µg of total RNA.

Il-21/IL-22 mRNA ratio was the highest in the degree of differentiation G1 (Figure 2). In all cancer tissues, regardless of degree of differentiation, the value of Il-21/IL-22 mRNA ratio was higher comparing to a control group (respectively: G1 vs. C *p* = 0.003405; G2 vs. C *p* = 0.008114; G1 vs. C *p* = 0.007473). Moreover, statistically significant difference was found between G1 and G3 (*p* = 0.032391).

The concentration of Il-21 was determined in the serum and peritoneal fluid of women with ovarian cancer. As the obtained values did not correspond to the normal distribution, the results were presented in the form of the median and the lower and upper interquartile range (Q1 and Q3).

In the serum of women with ovarian cancer, Q1 and Q3 were respectively: 314.25 and 736.00 with a median of 408.68 pg/mL. A statistically significantly lower concentration of IL-21 in the serum of women with ovarian cancer was demonstrated compared to the concentration in the control group (*p* < 0.001), where Q1 and Q3 were respectively: 722.19 and 909.60 with a median of 793.59 pg/mL.

In the peritoneal fluid of women with ovarian cancer, Q1 and Q3 were: 113.36 and 356.13, respectively, with a median of 175.63 pg/mL. A statistically significant higher concentration of the examined parameter was found in the serum of women with ovarian cancer as compared to the concentration in the peritoneal fluid (*p* < 0.0001).

**Figure 3:**
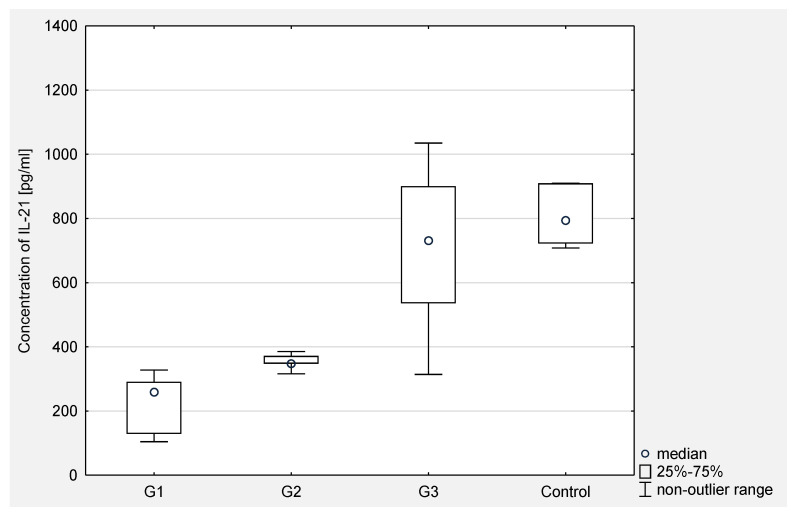
The concentration of IL-21 in the serum of women with ovarian cancer depending on the degree of differentiation G1 (well differentiated), G2 (moderately differentiated), G3 (poorly differentiated) compared to the control group.

**Figure 4:**
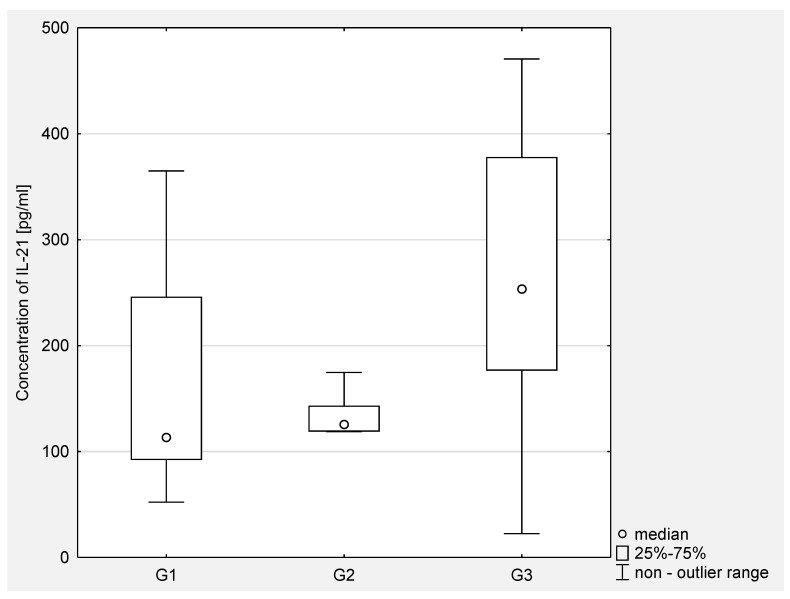
The concentration of IL-21 in the peritoneal fluid in women with ovarian cancer depending on the degree of differentiation G1 (well differentiated), G2 (moderately differentiated), G3 (poorly differentiated).

Then, the concentration of IL-21 in serum and the peritoneal fluid of women with ovarian cancer depending on the degree of histopathological differentiation of the cancer was analyzed. The analysis of the test results showed a statistical significance between G2 and G3 (*p* < 0.001) and between G1 and G3 (*p* < 0.0001) in the serum of the examined women. In the peritoneal fluid of women with ovarian cancer, a statistically significant difference was found only between G2 and G3 (*p* < 0.05). The obtained results are presented in Figure 3 and Figure 4.

The results of Il-22 concentration measurement in the serum and peritoneal fluid of women with ovarian cancer were presented in the form of the median and the lower and upper interquartile range (Q1 and Q3).

In the serum of women with ovarian cancer, Q1 and Q3 were respectively: 337.59 and 771.33 with a median of 572.64 pg/mL. A statistically significant higher concentration of IL-22 in the serum of women with ovarian cancer was demonstrated compared to the concentration in the control group (*p* < 0.001), where Q1 and Q3 were respectively: 66.01 and 140.04 with a median of 98.62 pg/mL.

In the peritoneal fluid of women with ovarian cancer, Q1 and Q3 were 132.40 and 275.70, respectively, with a median of 186.01 pg/mL. A statistically significant higher concentration of the examined parameter in the serum of women with ovarian cancer was demonstrated compared to the concentration in the peritoneal fluid (*p* < 0.0001).

Then, the concentration of IL-22 in serum and peritoneal fluid in women with ovarian cancer depending on the degree of histopathological differentiation of the cancer was analyzed. The analysis of the test results showed a statistical significance between G2 and G3 (*p* < 0.0001) and between G1 and G3 (*p* < 0.0001) in the serum of women. In the peritoneal fluid of women with ovarian cancer, no statistically significant differences were found between the degrees of histopathological differentiation of the cancer.

**Figure 5:**
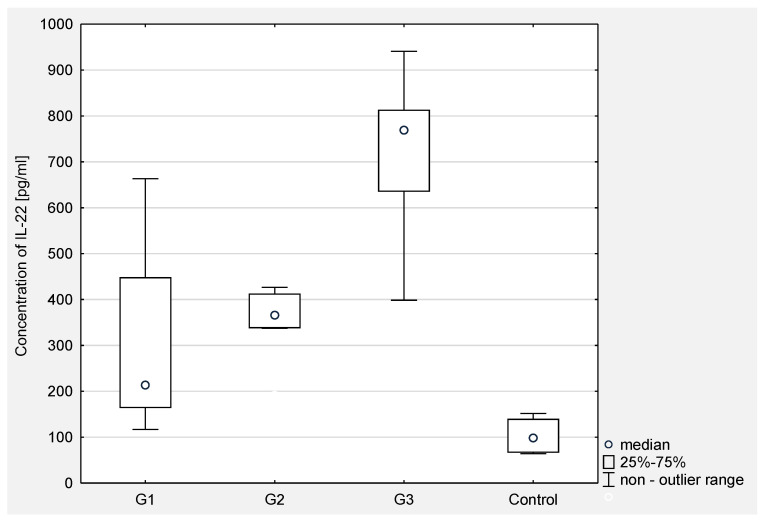
The concentration of IL-22 in the serum of women with ovarian cancer depending on the degree of differentiation G1 (well differentiated), G2 (moderately differentiated), G3 (poorly differentiated) compared to the control group.

**Figure 6:**
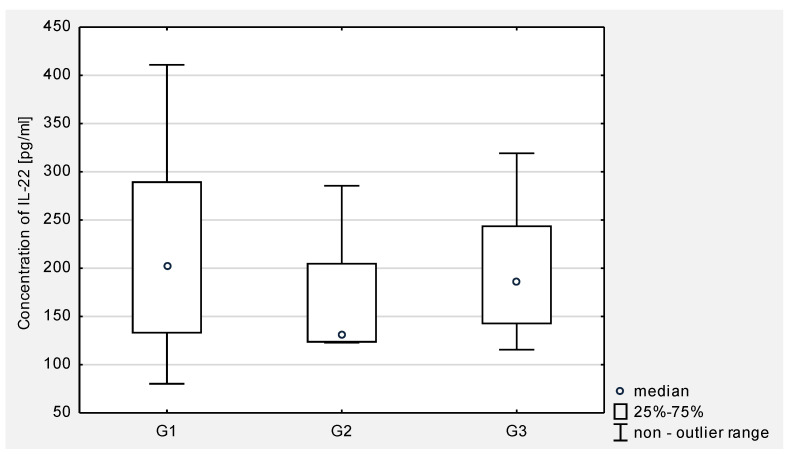
The concentration of IL-22 in the peritoneal fluid in women with ovarian cancer in relation to on the degree of differentiation G1 (well differentiated), G2 (moderately differentiated), G3 (poorly differentiated).

The obtained results are presented in Figure 5 and Figure 6.

**Figure 7:**
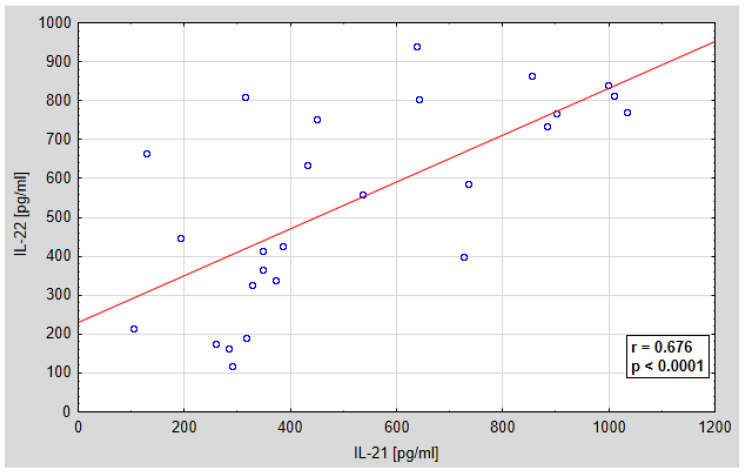
Linear regression curve showing the relationship between IL-21 and IL-22 levels in the serum of women with ovarian cancer; *r*-correlation coefficient, *p*-probability value.

**Figure 8:**
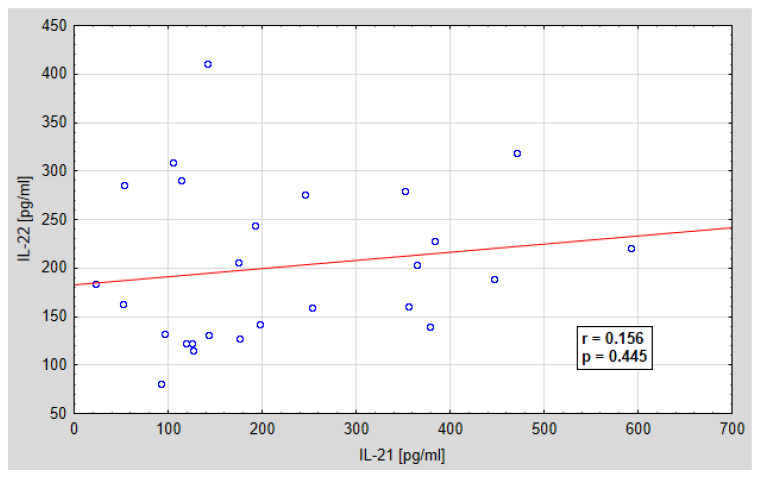
Linear regression curve showing the relationship between the concentration of Il-21 and IL-2 in the peritoneal fluid of women with ovarian cancer; *r*-correlation coefficient, *p*-probability value.

**Figure 9:**
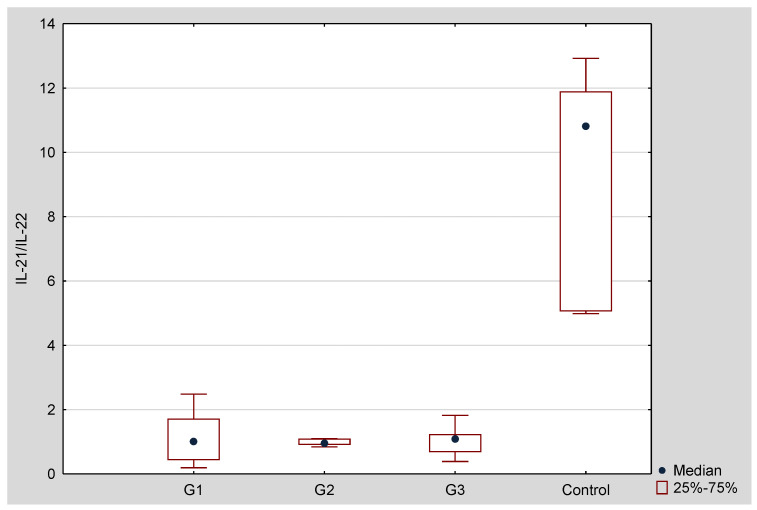
IL-21/IL-22 ratio in the serum of women with ovarian cancer depending on the degree of differentiation G1 (well differentiated), G2 (moderately differentiated), G3 (poorly differentiated) compared to the control group.

**Figure 10:**
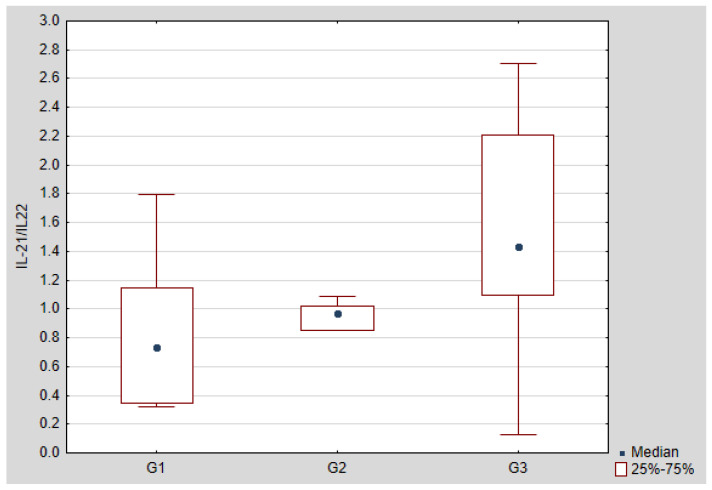
IL-21/IL-22 ratio in the peritoneal fluid of women with ovarian cancer depending on the degree of differentiation G1 (well differentiated), G2 (moderately differentiated), G3 (poorly differentiated).

Further analysis concerned the assessment of the relationship between the serum concentration of IL-21 and IL-22 and the peritoneal fluid concentration of IL-21 and IL-22. A statistically significant positive correlation was found between IL-21 and IL-22 in the serum of the examined women, and a positive, but not statistically significant correlation between the concentration of IL-21 and IL-22 in the peritoneal fluid of the examined women. The regression curves are shown in Figure 7 and Figure 8. Ratio of IL-21/IL-22 in serum and peritoneal fluid in patients with ovarian cancer is shown in Figure 9 and Figure 10.

## 4. Discussion

For years, the efforts of researchers have been focused on understanding the mechanisms of the formation and development of ovarian cancer in order to identify markers helpful not only in early diagnosis, but also in monitoring therapy. The challenge of contemporary gynecological oncology is to develop an effective targeted therapy that controls the expression of compounds that play a key role in the formation and development of the cancer. Numerous experimental studies and clinical observations indicate that chronic inflammation plays an important role in the pathogenesis of ovarian cancer, in which cells of the immune system participate, which, through secreted mediators in the tumor’s environment, participate in the anti-tumor response and may promote tumor growth, which may be manifested by changes in tissue expression, serum concentrations, and peritoneal fluid.

Recent studies have shown that interleukin 21 and interleukin 22 play an important role in the pathogenesis of cancer, which role in the formation and development of ovarian cancer is still not fully understood. Therefore, the aim of the analysis performed in the study was to assess the expression of IL-21 and IL-22 in the tumor tissue and the concentration of these parameters in the serum and peritoneal fluid in patients with ovarian serous cancer, taking into account the degrees of histological differentiation of the tumor.

For the first time, the expression of genes encoding IL-21 and IL-22 was assessed at the mRNA level in ovarian tumor specimens. The number of mRNA copies of the studied genes was determined by RT-qPCR method (quantitative method real-time). The studies showed that in the samples of the G1 histological differentiation, the number of mRNA copies of IL21 gene was statistically significantly higher, not only compared to the control group, but also compared to the G2 and G3 histological differentiation samples. In the case of IL22, we did not observe any differences in the number of mRNA copies between the cancer specimens and the control group, as well as between the G1, G2, and G3 histologically differentiated groups. The studies by Fagerberg et al. [25] did not show the expression of IL21 and IL22 at the mRNA level in normal ovaries. Therefore, the presence of mRNA of these interleukins in the sections studied by us, both malignant and benign, may be caused by the presence of cells of the immune system. The differences observed by us suggest that the interaction with the cells of the immune system may have a different course in malignant and benign tumors, but this requires further detailed studies.

Our studies showed a statistically significantly lower concentration of IL-21 in the serum of women with ovarian cancer compared to the concentration in the control group, which indicates the participation of the tested cytokine in the immune response against ovarian cancer cells. In addition, statistical significance was demonstrated between the concentration of the tested cytokine and the degrees of histological differentiation in cancer: G2 and G3, and between G1 and G3, which indicates a relationship between IL-21 secretion and histological differentiation in cancer and may prove useful in the future in selecting the optimal therapeutic treatment and in the assessment of prognosis.

Interesting observations were also provided by the analysis of the concentration of IL-21 in the peritoneal fluid of the examined women. The concentration of this parameter was significantly lower in the peritoneal fluid as compared to the serum, which may indicate that the source of the tested cytokine in the serum may be cells of the immune system, which, through secreted mediators, show increased cytotoxic activity against cancer cells. Moreover, it was shown that the concentration of IL-21 in the peritoneal fluid of women with ovarian cancer differed between the grades of G2 and G3, which proves that the secretion of IL-21 is related to the histological differentiation of ovarian cancer also in this biological fluid.

So far, no studies have been conducted to evaluate the expression of IL-21 in tumor tissue and the concentration in serum and peritoneal fluid. However, the potential usefulness of using this cytokine in the diagnosis of ovarian cancer was analyzed. Such research was conducted by Chen YL. et al. [19], who showed the usefulness of the assessment of interleukins IL-17a and IL-21 as prognostic and therapeutic biomarkers. Studies on the role of IL-21 in the anti-tumor response were also conducted by Hermans et al. [26], who pointed to the therapeutic potential of the transient inhibition of LDH during adoptive T-cell immunotherapy with the anti-tumor effect of inhibiting LDH and IL-21. Similar studies were conducted by Dou et al. [27], who showed that SKOV3 cells genetically modified to secrete biologically active IL-21 and GM-CSF were effective in inducing anti-tumor immunity by increasing NK cytotoxicity, promoting the expression of MIC A/B, ICAM-1, and NKG2D molecules, as well as increasing IFN-γ and TNF-α in the nude mouse model. On the other hand, Bhatt et al. [28] have shown that IL-21 has a strong anti-tumor activity against mantle lymphoma (MCL) cells through direct cytotoxic and indirect immunological effects. According to the authors, in vivo treatment with IL-21 leads to complete regression of FC-muMCL1 tumor in syngeneic mice through NK and T cell dependent mechanisms. Similar studies were also conducted by Li et al. [29], who showed that IL-21 may affect T cells, which are involved in the anti-tumor response, by fusing with an anti-PD-1 antibody. Wang et al. [30] assessed the role of interleukin 21 and its receptor in proliferation, migration, and invasion of breast cancer cells. The conducted research has shown that IL-21 promotes proliferation, invasion and migration of IL-21R + MDA-231 breast cancer cells, it does not show such strong properties against MCF-7 and ZR-75.1 cells, in which IL-21R expression is weak or negative. Moreover, the role of IL-21R in signaling pathways of matrix metalloproteinases, which are necessary for the processes of migration of MDA-231 cells, has been demonstrated. Zhang et al. [31] showed that reduction of the tumor and increased survival is accompanied by an increase of IFN-γ, IL-21, and TNF-α in serum, as well as the cytotoxic activity of the spleen. On the other hand, Gu et al. [32] showed that the co-expression of two members of the γ chain family of the cytokine receptor, IL-21 and IL-7, in anti-cancer vaccines increases anti-tumor immunity in a CD4 + and CD8 + T cell-dependent manner and generates an effective immune memory.

The increase of IL-21 mRNA in tumor tissue at the G1 stage may indicate that there are still efficient mechanisms aimed at limiting its growth, which is also reflected in the IL21/IL22 mRNA ratio value. In the G2 and G3 stages, the expression is reduced in tumor tissue, possibly as a result of disturbed intracellular signaling or epigenetic changes.

On the other hand, the increase in protein concentration, both in the serum and in the peritoneal fluid, suggests the activation of systemic mechanisms and a strong recruitment of the immune system. Due to blocked mechanisms in the tumor tissue itself, the antitumor effect of the immune system is probably ineffective, despite its strong stimulation. However, this hypothesis requires further investigation. It should also be taken into account that gene expression is a multi-step process and its regulation is complex. Strongly activated gene transcription in the tumor tissue does not mean that protein is synthesized and secreted. In our research, the protein source are likely cells of the immune system. If tumor tissue were the main source of interleukins, the protein concentration would be higher in the peritoneal fluid than in the serum.

An interesting observation is the fact that serum protein concentration is significantly decreased by the G1 stage and the concentration increases with the degree of histological differentiation of the cancer. In the G3 stage, the IL21 concentration increases to a level comparable to that in the control. Most likely, in the lower degree of histological differentiation of the cancer, the mechanisms responsible for “masking” the presence of cancer cells are activated. In our research, the study group was not very large, so the results should be interpreted with caution.

Further analysis included the evaluation of interleukin 22 expression at the mRNA level in tumor tissue and serum and peritoneal fluid concentration in women with ovarian cancer. The studies conducted so far have shown that the role of the interleukin IL-22 is complex. On the one hand, this cytokine promotes tumor development, is responsible in particular for the proliferation of malignant neoplasm of epithelial origin, but on the other hand, the intensification of its secretion may constitute a barrier against tumor development by suppressing inflammation due to the functions that protect against tissue damage, promote tissue repair, and prevent inflammation [33,34]. Our studies showed that the concentration of IL-22 in the serum of women with ovarian cancer was statistically significantly higher than that in the control group, which proves the participation of this cytokine in pro-cancer immune response through direct and indirect influence involving the immune system cells participating in the regulation of inflammation. In addition, a statistically significant higher concentration of the parameter in the serum of women with ovarian cancer was demonstrated compared to the concentration in the peritoneal fluid, which indicates increased secretion of Il-22 in the systemic immune response. Moreover, a relationship has been demonstrated between the concentration of IL-22 and the degrees of histological differentiation of cancer, including: G2 and G3, and between G1 and G3 in the serum of women, which indicates the relationship of this cytokine with tumor development and may in the future prove useful in selecting the optimal therapeutic treatment and in assessing the prognosis.

Further analysis concerned the assessment of the relationship between the concentration of IL-21 and IL-22 in the serum and in the peritoneal fluid. The demonstrated correlation between the concentrations of the tested parameters, both in the serum and in the peritoneal fluid, may indicate the need for further research in order to create complex diagnostic and therapeutic strategies targeting many different mechanisms of the immune response. Due to the fact that the interactions between these parameters may affect the direction of their biological activity, the stoichiometric relationship between the concentration of IL-21 and IL-22 and the number of IL21/IL22 mRNA copies was assessed. The determined IL-21/IL-22 ratio, both at the mRNA level in the tissue and at the protein level in the serum and in the peritoneal fluid in patients with ovarian cancer, showed a variable share of the tested cytokines in the IL-21/IL-22 system, depending on the histological differentiation of the cancer.

Similar studies were conducted by Balint et al. [20], who assessed the expression of IL-22 in human ovarian cancer tissues and in ascites samples. Expression of both IL-22 and the IL-22 receptor was higher in cancer tissues compared to the control tissue, which correlated with poor prognosis. Studies have shown that interleukin-22 is an important factor in the ovarian cancer tumor microenvironment because it stimulates tumor growth by increasing proliferation and serves as a protective factor for ovarian cancer during TNF-induced apoptosis. According to the authors, IL-22 is a potential therapeutic target and/or biomarker in human ovarian cancer. Wang et al. [35] showed an increased expression of Th22 lymphocytes, which produce IL-22 in the tumor microenvironment, which, in their opinion, may stimulate tumor growth and affect the patient’s further prognosis. Katara et al. [36] demonstrated the presence of IL-22 at all stages of the breast cancer, and that blocking of IL-22 gene expression resulted in inhibition of tumor invasion and reduction of metastasis. On the other hand, Rui et al. [37] showed an increased expression of the interleukin-22 receptor 1 in breast cancer. IL-22 binds to the IL-10R2 and IL-22R1 receptor complexes, activating the transcription factor STAT3, thereby promoting tumor progression. IL-22R1 is expressed exclusively on the surface of epithelial and tissue cells. Moreover, IL-22 promoted the expression of pro-tumor HOXB-AS5 proteins in breast cancer. Khosravi et al. [38] showed that high expression of IL-22RA1 in KRAS mutated lung adenocarcinoma is associated with a short remission time. Genetic ablation of IL-22 resulted in a significant decrease in tumor mass and a reduction in the number of tumor cells, their proliferation, and the ability to activate STAT3, and it was also associated with a reduction in angiogenesis and the number of inflammatory cells that infiltrated the lung tissue.

## 5. Conclusions

Local and systemic changes in the immune system with the participation of soluble mediators IL-21 and IL-22 indicate the participation of these parameters in the development of ovarian cancer and may participate in pro and anti-inflammatory activation involving the tested cytokines.

Modulation in the IL-21 and IL-22 system may affect the course of the inflammatory process that accompanies the development of cancer, which may prove useful in the development of new diagnostic and therapeutic strategies used in patients with ovarian cancer, which requires further studies.

## Data Availability

Data is contained in the article.

## Supplementary Materials

The following are available online at https://www.mdpi.com/article/10.3390/jcm10143058/s1, Figure S1: Quality of total RNA, Bioanalyzer Agilent electrophoresis run, Figure S2: Melting peak analysis showing specificity of RT-qPCR reaction, Figure S3: Agarose gel electrophoresis of IL22 PCR-amplified product, Figure S4: Agarose gel electrophoresis of IL21 PCR-amplified product.
