# Supplementary material for: The Assessment of IL-21 and IL-22 at the mRNA Level in Tumor Tissue and Protein Concentration in Serum and Peritoneal Fluid in Patients with Ovarian Cancer
Source: J Clin Med. 2021 Jul 9;10(14):3058. doi: 10.3390/jcm10143058 (PMC8304053; doi:10.3390/jcm10143058)
Supplement: Supplementary file 1 [file jcm-10-03058-s001.zip › jcm-1276532-supplementary.pdf]

Supplementary Material

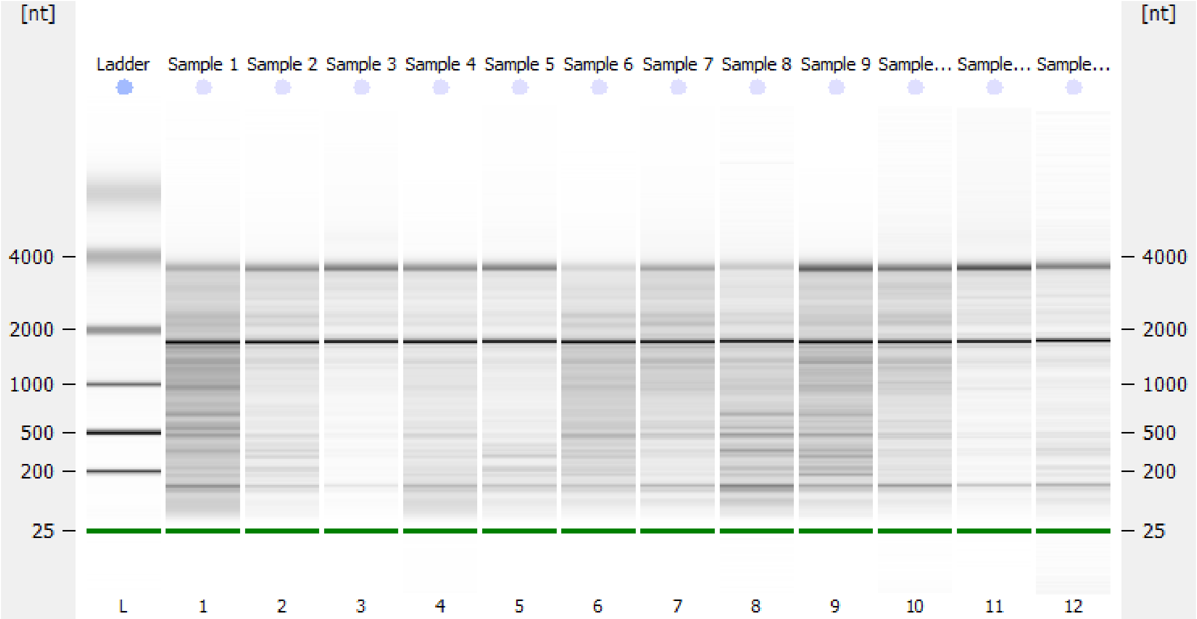

Figure S1. Quality of total RNA, Bioanalyzer Agilent electrophoresis run, lane 1 : the RNA ladder (RNA 6000 Nano Marker, Agilent Technologies), lane 2-12 : total RNA image after isolation with Trizol Reagent.

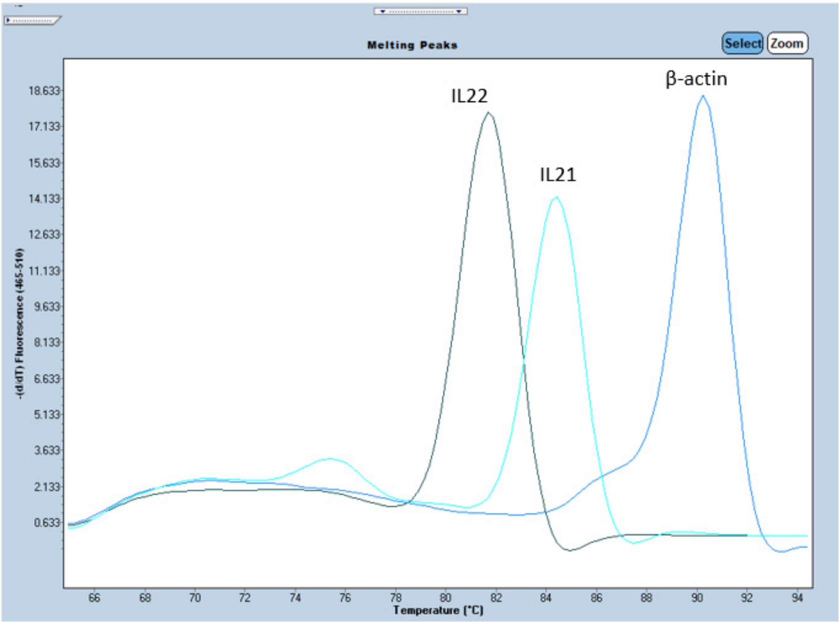

Figure S2. Melting peak analysis showing specificity of RT-qPCR reaction (LightCycler® 480 System, Roche, Switzerland).

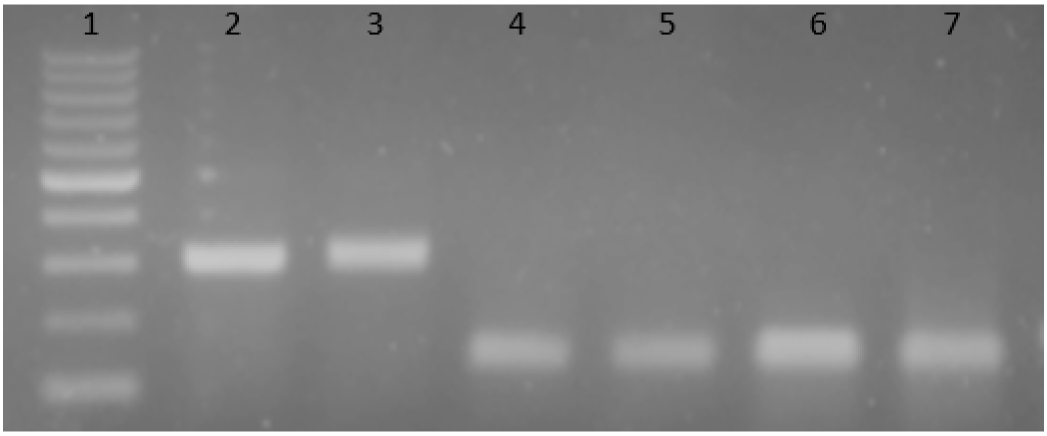

Figure S3. Agarose gel electrophoresis of IL22 PCR-amplified product. Lane 1: DNA lader 100-1000 bp (DNA Marker1 100-1000 pz, A&A BIOTECHNOLOGY, Poland); lane 2-3: β-actin PCR-amplified product (295 bp); lane 4-7: IL22 PCR-amplified product (148 bp).

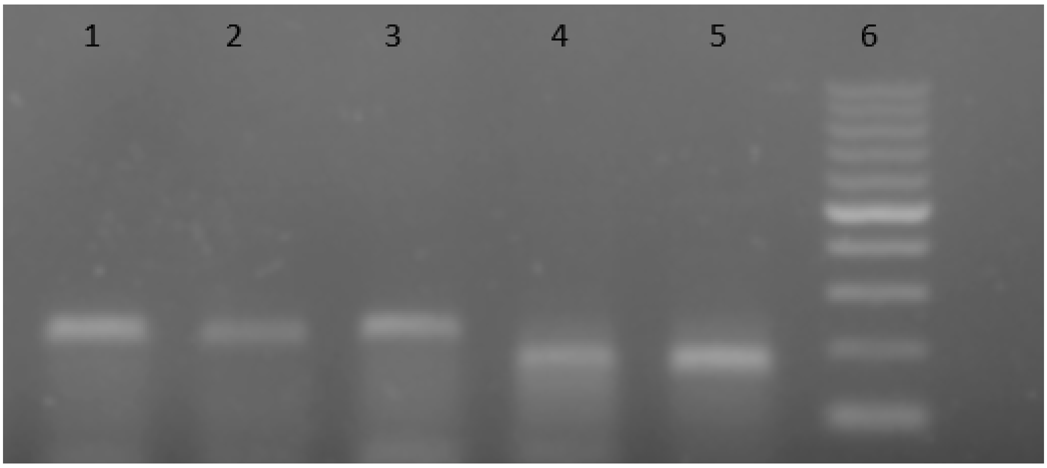

Figure S4. Agarose gel electrophoresis of IL21 PCR-amplified product. Lane 1-3: IL21 PCR-amplified product (221 bp); lane 4-5 FLT1 PCR-amplified product (182 bp; data not included in the study); lane 6: DNA lader 100-1000 bp (DNA Marker1 100-1000 pz, A&A BIOTECHNOLOGY, Poland).
